# Managing biological complexity across orthologs with a visual knowledgebase of documented biomolecular interactions

**DOI:** 10.1038/srep01011

**Published:** 2012-12-20

**Authors:** Vincent VanBuren, Hailin Chen

**Affiliations:** 1 Texas A&M HSC College of Medicine, Systems Biology and Translational Medicine

## Abstract

The complexity of biomolecular interactions and influences is a major obstacle to their comprehension and elucidation. Visualizing knowledge of biomolecular interactions increases comprehension and facilitates the development of new hypotheses. The rapidly changing landscape of high-content experimental results also presents a challenge for the maintenance of comprehensive knowledgebases. Distributing the responsibility for maintenance of a knowledgebase to a community of subject matter experts is an effective strategy for large, complex and rapidly changing knowledgebases. Cognoscente serves these needs by building visualizations for queries of biomolecular interactions on demand, by managing the complexity of those visualizations, and by crowdsourcing to promote the incorporation of current knowledge from the literature.

Imputing functional associations between biomolecules and imputing directionality of regulation for those predictions each require a corpus of existing knowledge as a framework to build upon. Comprehension of the complexity of this corpus of knowledge will be facilitated by effective visualizations of the corresponding biomolecular interaction networks. **Cognoscente** (http://vanburenlab.medicine.tamhsc.edu/cognoscente.html) was designed and implemented to serve these roles as a knowledgebase and as an effective visualization tool for systems biology research and education. **Cognoscente** currently contains over 413,000 documented interactions, with coverage across multiple species. Perl, HTML, **GraphViz**^1^, and a **MySQL** database were used in the development of **Cognoscente**.

**Cognoscente** was motivated by the need to (1) update the knowledgebase of biomolecular interactions at the user level, and (2) flexibly visualize multi-molecule query results for heterogeneous interaction types across different orthologs. Satisfying these needs provides a strong foundation for developing new hypotheses about regulatory and metabolic pathway topologies. Several existing tools provide functions that are similar to **Cognoscente**, so we selected several popular alternatives to assess how their feature sets compare with **Cognoscente** (Table 1)^2^,^3^,^4^,^5^,^6^,^7^,^8^,^9^,^10^,^11^. All databases assessed had easily traceable documentation for each interaction, and included protein-protein interactions in the database. Most databases, with the exception of **BIND**, provide an open-access database that can be downloaded as a whole. Most databases, with the exceptions of **EcoCyc** and **HPRD**, provide support for multiple organisms. Most databases support web services for interacting with the database contents programmatically, whereas this is a planned feature for **Cognoscente**. **MINT**, **STRING**, **IntAct**, **EcoCyc**, **DIP** and **Cognoscente** provide built-in visualizations of query results, which we consider among the most important features for facilitating comprehension of query results. **BIND** supports visualizations via **Cytoscape**^12^,^13^. **Cognoscente** is among a few other tools that support multiple organisms in the same query, protein->DNA interactions, and multi-molecule queries. **Cognoscente** has planned support for small molecule interactants (i.e. pharmacological agents). **MINT**, **STRING**, and **IntAct** provide a prediction (i.e. score) of functional associations, whereas **Cognoscente** does not currently support this. **Cognoscente** provides support for multiple edge encodings to visualize different types of interactions in the same display, a crowdsourcing web portal that allows users to submit interactions that are then automatically incorporated in the knowledgebase, and displays orthologs as compound nodes to provide clues about potential orthologous interactions. The main strengths of **Cognoscente** are that (1) it provides a combined feature set that is superior to any existing database, and (2) that it provides a unique visualization feature for orthologous molecules, and relatively unique support for multiple edge encodings, crowdsourcing, and connectivity parameterization. The current weaknesses of **Cognoscente** relative to these other tools are (1) that it does not fully support web service interactions with the database, (2) it does not fully support small molecule interactants, and (3) it does not score interactions to predict functional associations. Web services and support for small molecule interactants are currently under development.

## Results

### Crowdsourcing

**Figure 1:**
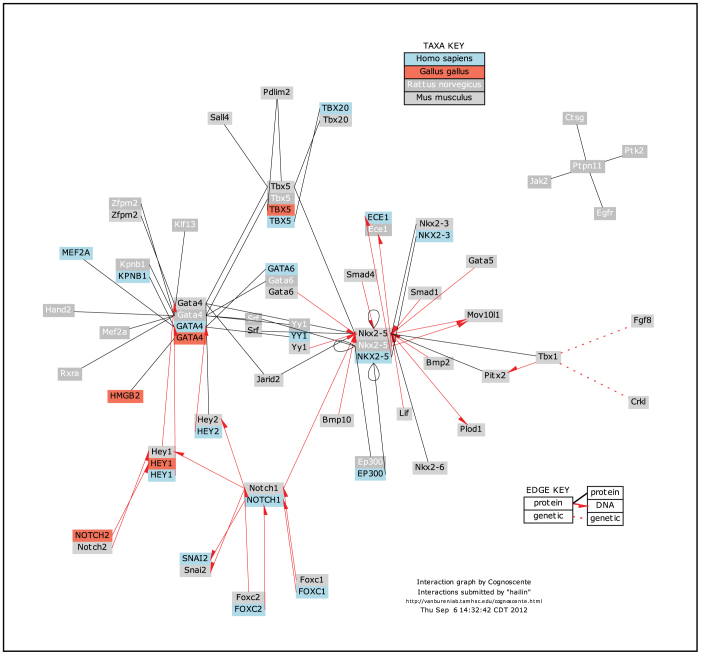
Biomolecular interactions manually added to Cognoscente via a web-based submission portal. Interactions supplied by NCBI Interaction GeneRIFs were found to be incomplete with respect to documented interactions. We subsequently identified 75 interactions relevant to cardiac development in the literature, and manually added them to Cognoscente via a web-based submission portal.

**Figure 2:**
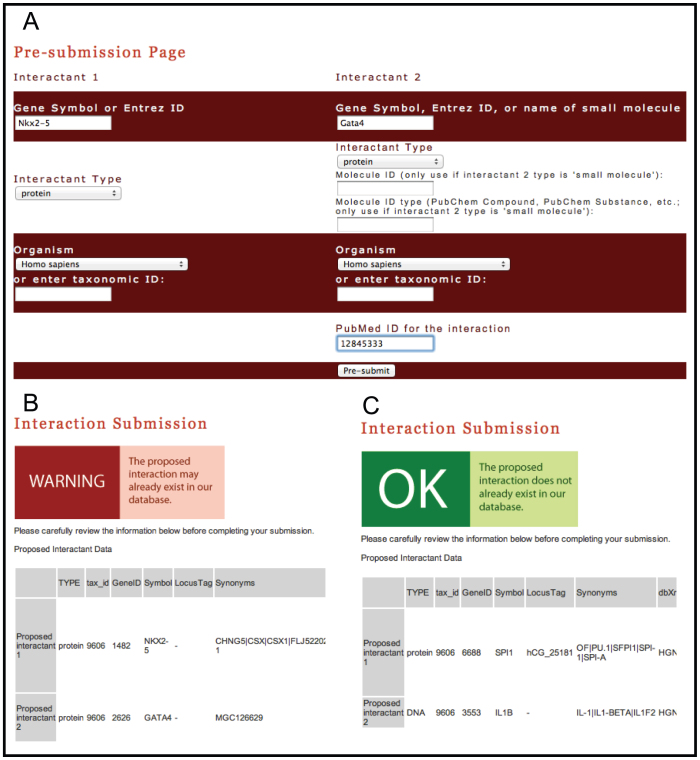
Submitting new biomolecular interactions in Cognoscente. User submissions interface for **Cognoscente** (A). Feedback is provided to the user about their proposed submission. If the submission appears to be redundant with an entry in the knowledgebase, the user receives a warning about the potential redundancy (B). Likewise, users receive a ‘green light’ if the proposed interaction does not already exist (C). In addition to these notifications, the system also retrieves gene information based on the user-supplied gene IDs, and retrieves the entire citation and abstract for the submitted PubMed IDs. These measures are expected to reduce redundancy and errors due in manual submissions.

**Cognoscente** was partly motivated by incomplete coverage of documented biomolecular interactions in existing databases. In the first iteration of **Cognoscente**, which contained over 270,000 interactions built from Interaction GeneRIFs compiled by NCBI^8^, we noted that that at least 75 interactions relevant to early cardiac development were absent. We have thus added 75 interactions relevant to cardiac development that we manually collected from the literature (Fig. 1). These interactions were not in the databases above, and were discovered through a manual search of the PubMed database for documented interactions with selected genes. This demonstrates that the NCBI Interaction GeneRIFs do not provide a complete catalogue of documented biomolecular interactions, and thus motivates a mechanism for user submissions. To encourage accurate knowledgebase submissions, users are asked to pre-submit interactions so that the submission software module can check for redundancies and report back to the user with the details about their submissions (Fig. 2). This will reduce errors and redundancies in new knowledgebase submissions. Accountability and credit for new submissions is afforded by requiring that each submitter registers for an account. Each submission is tagged with the submitter's name, and submitter names are shown in any query results that include that submitted entry.

### Search by gene symbol or Gene ID

**Figure 3:**
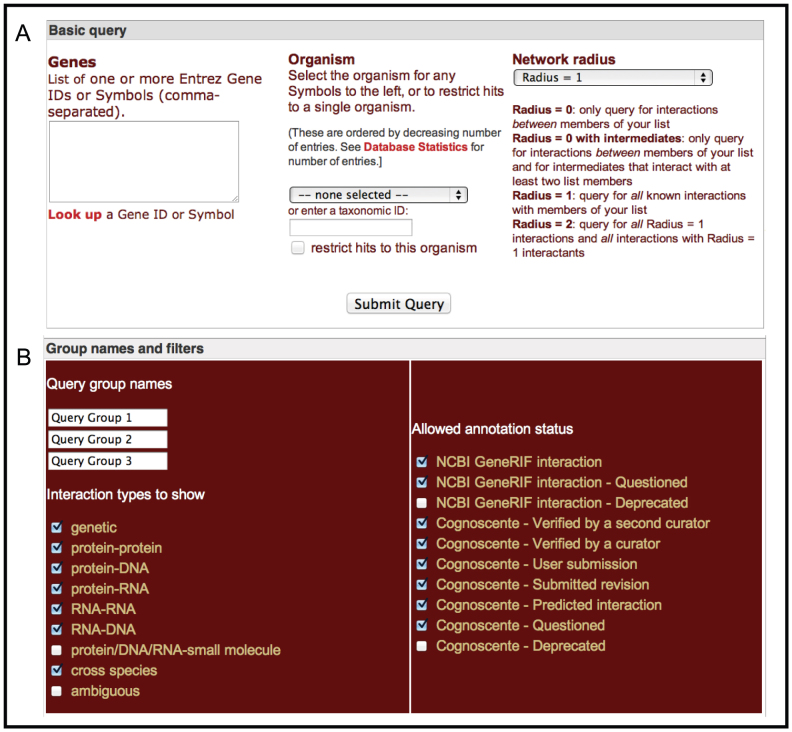
The Basic Query interface for Cognoscente allows queries of multiple genes, the option to show orthologous interactions, and an option to prescribe the complexity of the network to draw in the result (A). A list of multiple genes can be divided into groups by separating the entered gene symbols with a forward slash (‘/’). In the results, the queried genes will be indicated as groups by different colored boxes around the network nodes. These groups (up to 3) may be named (B), which will be indicated in an automatically generated legend in the resulting graph. Results may be filtered by interaction type or by annotation status (B).

**Cognoscente** queries are conducted using Entrez Gene IDs or Entrez Symbols (Fig. 3). Unofficial gene synonyms are permitted in queries, as long as the synonyms unambiguously match the official gene symbols. Queries with Gene Symbols or unofficial synonyms require specifying the organism associated with the respective symbols.

### Generation of GraphViz dot files for rendering networks

**Figure 4:**
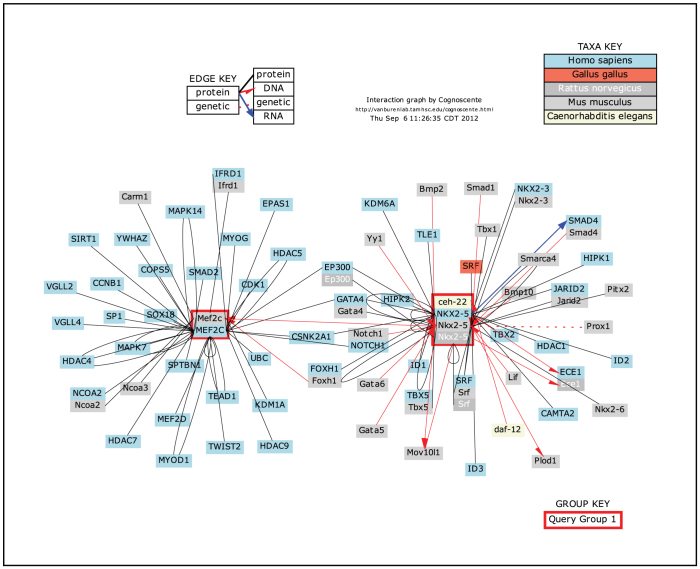
Graphical query result for human genes ‘*Nkx2-5*, *Mef2c*’ in Cognoscente. This query was parameterized to search across all known orthologs for all primary (*radius* = 1) biomolecular interactions with either *Nkx2-5* or *Mef2c*. **Cognoscente** automatically generates legends (as shown here) that are inserted into the graphic.

**GraphViz** is software that was developed at AT&T for drawing graphs defined in the dot scripting language. **Cognoscente** leverages **GraphViz** by automatically generating dot script files for arbitrary graphs, and runs the scripts in **GraphViz**, which defines the layout and performs rendering of the graphs (Fig. 4).

### Hyperlinks

Network specifications sent to **GraphViz** are configured to provide a mapping of nodes and edges for web interactivity, with mouse-over hints and links from the nodes to Entrez Gene and links from the edges to record pages containing interaction details and supporting references. The result page for a query in **Cognoscente** contains a rendered PNG version of the graph with mapped outlinks, the text of the dot file used to create that rendering (so the user can modify the dot file and create their own rendering with **GraphViz**, if needed), and a link to a high-quality PDF of the graph.

### Hypernodes

Nodes in **Cognoscente** are hypernodes, meaning that each representation as a biomolecule (i.e. DNA, RNA, protein) is dependent on the type of edge that connects the node to another node. For example, a black line indicates a protein-protein interaction, and a red arrow indicates a protein->DNA interaction. A node *X* that has both a black line connecting it to node *Y* and a red arrow pointing to it from node *Z* is thus representing both the protein *X* and the DNA of gene *X*, depending on the interaction represented.

### Orthology

**Cognoscente** renders networks that contain nodes that are stacked by orthology. The corpus of knowledge of biomolecular interactions differs in coverage for different model organisms. Organizing orthology as stacked nodes thus presents biomolecular interaction knowledge in a manner that facilitates comparisons between orthologs, and interactions known in one organism may provide clues to complement knowledge in another organism. The default behavior of **Cognoscente** will return results that use stacked nodes to represent orthology. However, the user can choose to confine results to a single organism.

### Lookup integration with StarNet

**Figure 5:**
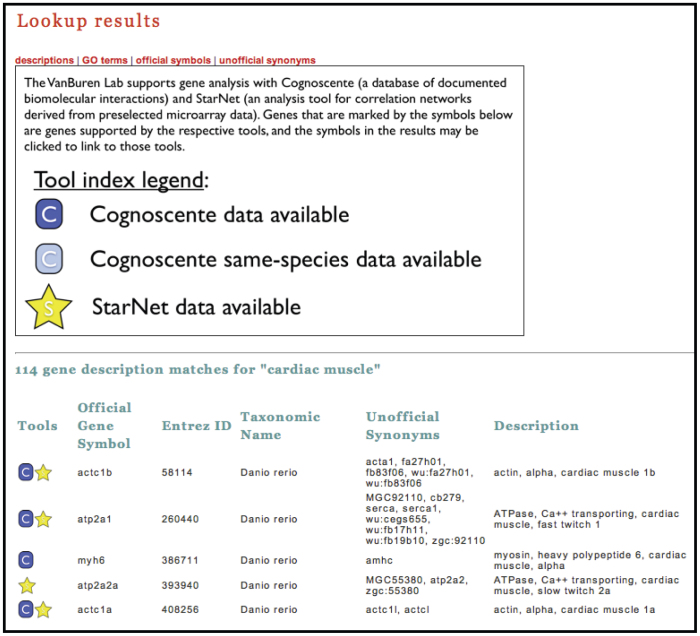
Partial results for the query “cardiac muscle” using the Gene Lookup tool (http://vanburenlab.medicine.tamhsc.edu/gene_lookup.html). Full results include a search against gene descriptions, GO terms, official gene symbols and unofficial gene synonyms. Graphic symbols (as described in the legend) appear next to genes that are supported by StarNet or Cognoscente, respectively. These graphics may be clicked by the user to redirect to the appropriate query page, and automatically populate parameters on the query page for the respective gene.

There is a lack of knowledge about biomolecular interactions for many genes and gene products. Users of **Cognoscente** may thus query a gene for which there are no results. To expedite the use of **Cognoscente**, as well as to provide users a way to look up unfamiliar gene symbols, we created a Gene Lookup Tool (http://vanburenlab.medicine.tamhsc.edu/gene_lookup.html). This tool allows a keyword search that simultaneously searches Gene Descriptions, Gene Ontology (GO) Terms, official Gene Symbols, and unofficial Gene Synonyms (Fig. 5). Queries with this tool return all Entrez Gene entries with a keyword match, and icons next to each matched entry indicate support by either **StarNet**^14^,^15^ (a yellow star) or **Cognoscente** (a white ‘C’ on a blue background). Clicking on one of these icons redirects the user to the respective tool and populates the parameters of that tool to query the matched Entrez Gene entry.

### Network radius

Documented connectivity among genes and gene products produces networks that are scale-free. This means that most nodes have low connectivity, but a few nodes have very high connectivity. To accommodate these variations in complexity, different ‘network radii’ can be drawn with **Cognoscente**. The four options available are *radius* = 0, *radius* = 0 *with intermediates*, *radius* = 1, and *radius* = 2, in order of increasing complexity of the resultant network. Here radius is defined as the prescribed graph distance with respect to each query gene. In ***radius* = 0** networks, only interactions between members of the query list are shown. In ***radius* = 0 *with intermediates*** networks, only interactions between members of the query list and intermediates that interact with at least two list members are shown. In ***radius* = 1** networks, all known interactions with members of the query list are shown. Finally, in ***radius* = 2** networks, all ***radius* = 1** interactions and all interactions with ***radius* = 1** interactants are shown. As we define it, *network radius* is different from the graph theoretic concept of radius (which applies to distances in an entire graph), as our definition is with respect to the query genes and their documented interactions. For multiple query genes, the graph theoretic radius can thus be higher than the prescribed network radius because multiple graphs (one for each query gene) are combined in a composite graph. Likewise, the graph theoretic radius can be smaller than the prescribed network radius where there are no documented interactions for a query gene.

### Filtering queries

In the ‘Group Names and Filters’ panel of the **Cognoscente** interface, users can exclude different types of interactions, or exclude entries based on annotation status. For example, a user can configure a query to only return results for protein-protein interactions, or only genetic interactions, or some arbitrary combination of interaction types.

### Multi-gene searches

Queries in **Cognoscente** may be composed of one or more genes, and results are merged into a single graph. This offers great flexibility and power for composing queries that bridge multiple pathways. One useful application of **Cognoscente** is that queries of selected gene sets (that may be either functionally or phenotypically related) can be used to reveal interacting intermediates between two genes/gene products of interest for further study.

### Query groups

A comma-separated list of query genes will be treated as a single ‘query group’. However, up to three different query groups may be specified by using a forward-slash (‘/’) separator between query groups. This has the effect of color-coding the query group members in the results, and indicating this color-coding in a legend. The default group names (‘Query Group 1’, ‘Query Group 2’, and ‘Query Group 3’) may be changed in the ‘Group Names and Filters’ panel of the **Cognoscente** interface.

### Managing complexity

**Figure 6:**
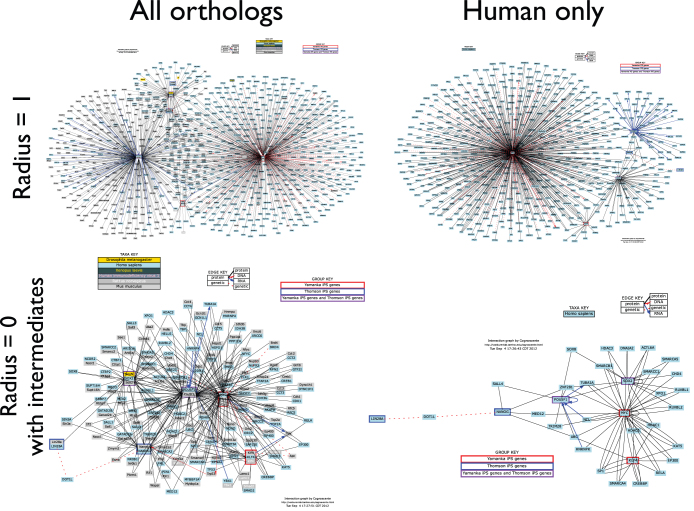
Managing complexity with Cognoscente. iPSC genes *Oct4* (*Pou5f1*), *Sox2*, *c-Myc* (*Myc*), *Klf4*, *Nanog*, and *Lin28A* were queried with **Cognoscente** using different approaches. In the upper left panel, all primary (*radius* = 1) interactions are shown across all orthologs. In the upper right panel, only *human* primary interactions are shown. In the lower left panel, *radius* = 0 interactions (those between members of the query list) and ‘intermediates’ (those where the interactant has interactions with at least two members of the query list) are shown across all orthologs. Finally, in the lower right panel, only *human* *radius* = 0 *interactions with intermediates* are shown, which eliminates hundreds of nodes as compared with the upper left panel.

Although visualizations of complex knowledge leverage our ability to comprehend visual complexity, this ability has limitations that vary with the context of the knowledge. Comprehension may thus be aided by exercising some control over the amount of complexity expressed in a visualization. The most obvious way of controlling complexity in Cognoscente visualizations is to query only one or a few genes rather than many genes. Complexity in Cognoscente visualizations is also parameterized in two main ways: (1) by either choosing to query all orthologs or confining the search to a single organism, and (2) by prescribing the network radius to retrieve in the query. This is illustrated with an example query for genes that were found to be important for the specification of inducible pluripotent stem cells (iPSCs). Induction of stem cells from human adult cells was first achieved in 2007^16^,^17^. Yamanaka and colleagues induced transformation into pluripotent stems cells using Oct4, Sox2, Klf4, and c-Myc^16^, whereas Thomson and colleagues used Oct4, Sox2, Nanog and Lin28A^17^. We queried “Oct4, Sox2, Klf4, Myc/Oct4, Sox2, Nanog, Lin28A” in Cognoscente, and varied the parameterization of complexity in four ways to demonstrate how visual complexity may be controlled in query results (Fig. 6). In the first query, all primary (*radius* = 1) interactants are visualized across orthologs, yielding a relatively complex graph with hundreds of nodes. This complexity is greatly reduced by constraining results to one species (*Homo sapiens*) and by constraining the visualized interactions to those between members of the query list and to those with interactants that have interactions with at least two members of the query list (our definition of ‘intermediate’). This reduces the number of nodes in the resulting network from several hundred to several dozen, and thus greatly increases comprehensibility. However, the more complex modalities for graphing these query results may also be desirable for their greater completeness, or in contexts where there are a smaller number of documented interactions for a given query.

### Future software maintenance

**Cognoscente** is maintained, upgraded and updated on an ongoing basis. Two main strategies are used to promote the sustainability of the knowledgebase. These are (1) automatically leveraging the Interaction GeneRIFs using a script that identifies new GeneRIFs catalogued by NCBI, processes those interactions, and adds them to the knowledgebase on a weekly schedule, and (2) providing a web portal for user submissions of interactions to complement Interaction GeneRIFs. These strategies were selected because they require very little in the way of centralized maintenance and are low cost, and together work to ensure that the knowledgebase is kept up-to-date. We plan to add web services to allow programmatic interactions with Cognoscente, and add support for drug and target interactions.

### Data access

The **Cognoscente** web application can be accessed at http://vanburenlab.medicine.tamhsc.edu/cognoscente.html. **Cognoscente** usage is freely licensed without warranty. Downloading the **Cognoscente** interaction table is freely available under the Creative Commons Attribution 3.0 Unported License (CC-BY-3.0) license at http://vanburenlab.medicine.tamhsc.edu/cognoscente_downloads.shtml. The downloadable interaction table is updated weekly, and an archive of previous versions is maintained at this site.

## Discussion

There are four main ways that we envision Cognoscente to be leveraged by biomedical scientists: 1. as a tool for quickly retrieving known interactions across orthologs for a particular gene, 2. to help interpret the results of high-content data approaches such as microarray analysis, 3. to build interactomes for arbitrary lists of genes, and 4. to aid the inference of biomolecular pathways for regulation and signaling. Supplementary Figure S1 shows an example of using **Cognoscente** to aid the interpretation of high-content data. Briefly, we retrieved the list of differentially expressed genes and a list of TRAP-ranked regulators from Kwong and colleagues’ recent melanoma study (Tables S1 and S5, respectively, from their publication)^18^. These genes were queried in **Cognoscente** in groups labeled ‘Up with respect to control’, ‘Down with respect to control’, and ‘TRAP-ranked regulators’, parameterized with *radius* = 0 *with intermediates*. The resultant graph reveals clues about potential regulatory relationships that induce the observed differential expressions (Supplementary Fig. S1). Supplementary Figures S2 and S3 show an example of using **Cognoscente** to build an arbitrary interactome. The mechanisms by which alcohol consumption inhibits bone healing after fracture are not well defined. We used the Lookup tool provided with **Cognoscente** to retrieve a list of all human genes annotated with a Gene Ontology (GO) term that contains the word “alcohol”, and another list of all human genes annotated with a GO term containing the word “bone” (Supplementary Table S1). These labeled groups were queried in Cognoscente and parameterized with *radius* = 0 and *radius* = 0 *with intermediates*, respectively (Supplementary Figs. S2 and S3). The results reveal connections between alcohol metabolism and bone metabolism that may be relevant to the effects of alcohol consumption on bone healing. The construction of arbitrary interactomes from genes implicated in a particular disease or developmental process will provide new hypotheses about intermediates that may also be relevant to the respective developmental process or disease (e.g. Fig. 6). These interactomes may in turn provide insights for the inference of relevant pathways.

We previously developed **StarNet**, which is a visual database for correlations in pre-selected microarray data^14^,^15^. **StarNet** allows queries of a single gene from one of ten organisms, and draws a correlation network radiating from that gene from precompiled microarray data. The future development of **Cognoscente** will include better integration with **StarNet**^14^,^15^, including overlay of known correlations in the interaction map, and inferential modeling combining interaction knowledge from **Cognoscente** and expression correlations from **StarNet**. This improved integration will allow users to directly combine interaction knowledge and expression correlation data in models that infer causal relationships, thus creating specific well-formed hypotheses from existing knowledge and data.

## Methods

### Source knowledge

**Cognoscente** was built and is routinely updated by extracting knowledge from NCBI's Interaction GeneRIFs^8^. The bulk of the interactions collected from NCBI in **Cognoscente** were originally extracted from HPRD^6^, BIND^10^, BioGrid^2^, or EcoCyc^5^. All interactions in the **Cognoscente** knowledge base are supported by one or more PubMed references. Documented knowledge of interactions that are not already extracted by this method may be added at any time by registered users (see **Results**, *Crowdsourcing*).

### Database schema

The Cognoscente knowledgebase is presently maintained on the VanBuren Lab web server as a MySQL database. The main database table of interactions has fields based on the table of Interaction GeneRIFs downloaded from NCBI. The database field structure is identical to the fields in the downloadable table of interactions on the Cognoscente web site (see **Results**, *Data Access*). Other tables in the knowledgebase include a table for registered *users*, a *history* table for tracking user alterations to the database to allow for quality assurance checks and the ability to revert to an earlier state of the database, and the *gene_info* and *homologene* tables downloaded from NCBI.

### Program logic

The web interface was encoded with HTML and Javascript, and interacts with a Perl CGI script that calls the main logic script, which is also written in Perl. The main logic script calls directly on **MySQL** for database queries and on **GraphViz** to construct visualizations of graphs. Orthologies were defined in **Cognoscente** using **HomoloGene**^19^. For queries in **Cognoscente** that include orthologous genes (the default behavior), a lookup is performed for genes that have the same HomoloGene ID as each queried gene. Orthologous genes are shown in composite (stacked) nodes only when there is a documented interaction for those genes in the knowledgebase and that interaction will appear in the resultant graph. Graph edges in the web-rendered image and **Cognoscente** ID numbers in the respective table of interactions are linked to record pages for each interaction, which include citations and abstracts extracted from PubMed using **RefSense**^20^.

### Drawing graphs

Graphs are drawn in **Cognoscente** using **GraphViz**. The *sfdp* algorithm for layouts is used to produce graphs in GIF format coupled with CMAP mapping to provide clickable graphs in the web interface, and to produce PDFs of the graph.

## Supplementary Material

Supplementary Information Supplementary Figures

Supplementary Information Supplementary Table S1

## Figures and Tables

**Table 1:** Features of biomolecular interaction databases

Interaction tool	Easily traceable documentation	Protein-protein interactions	Open access database can be downloaded in whole from web	Web services	Multiple Organisms	Network Visualization	Multiple organisms in same query	Clickable nodes and edges for details	Protein->DNA interactions	Small molecule interactions	Genetic Interactions	Multi-molecule queries	Predicts functional associations	Multiple edge encodings	Crowdsourcing web portal	Connectivity Parameterization	Compound nodes for orthologous molecules	Total features
**MINT**	✓	✓	✓	✓	✓	✓	✓	✓				✓	✓			✓		11
**STRING**	✓	✓	✓	✓	✓	✓		✓				✓	✓	✓				10
**IntAct**	✓	✓	✓	✓	✓	✓	✓		✓	✓			✓					10
**BIND**	✓	✓		✓	✓	#	✓		✓	✓	✓	✓						9
**GenRIF Interactions**	✓	✓	✓	✓	✓				✓	✓	✓				✓			9
**EcoCyc**	✓	✓	✓	✓		✓		✓	✓	✓								8
**DIP**	✓	✓	✓		✓	✓	✓	✓										7
**BiooGrid**	✓	✓	✓	✓	✓		✓				✓							7
**HPRD**	✓	✓	✓	✓														4
**Cognoscente**	✓	✓	✓	‡	✓	✓	✓	✓	✓	‡	✓	✓		✓	✓	✓	✓	14

^‡^Planned feature.

#Via Cytoscape.

## References

[b1] Gansner E. & North S. An open graph visualization system and its applications to software engineering. Softw. Pract. Exper. 00, 1–5 (1999).

[b2] Breitkreutz B. J. *et al.* The BioGRID Interaction Database: 2008 update. Nucleic Acids Res 36, D637–640 (2008). 18000002 10.1093/nar/gkm1001 PMC2238873

[b3] Jensen L. J. *et al.* STRING 8--a global view on proteins and their functional interactions in 630 organisms. Nucleic Acids Res 37, D412–416 (2009). 18940858 10.1093/nar/gkn760 PMC2686466

[b4] Kerrien S. *et al.* The IntAct molecular interaction database in 2012. Nucleic Acids Res 40, D841–846 (2012). 22121220 10.1093/nar/gkr1088 PMC3245075

[b5] Keseler I. M. *et al.* EcoCyc: a comprehensive view of Escherichia coli biology. Nucleic Acids Res 37, D464–470 (2009). 18974181 10.1093/nar/gkn751 PMC2686493

[b6] Keshava Prasad T. S. *et al.* Human Protein Reference Database--2009 update. Nucleic Acids Res 37, D767–772 (2009). 18988627 10.1093/nar/gkn892 PMC2686490

[b7] Licata L. *et al.* MINT, the molecular interaction database: 2012 update. Nucleic Acids Res 40, D857–861 (2012). 22096227 10.1093/nar/gkr930 PMC3244991

[b8] NCBI. GeneRIF: Gene Reference Into Function, <http://www.ncbi.nlm.nih.gov/gene/about-generif> (2012).

[b9] von Mering C. *et al.* STRING: a database of predicted functional associations between proteins. Nucleic Acids Res 31, 258–261 (2003). 12519996 10.1093/nar/gkg034 PMC165481

[b10] Willis R. C. & Hogue C. W. Searching, viewing, and visualizing data in the Biomolecular Interaction Network Database (BIND). Curr Protoc Bioinformatics **Chapter** 8, Unit 8 9 (2006 10.1002/0471250953.bi0809s12 18428770

[b11] Xenarios I. *et al.* DIP, the Database of Interacting Proteins: a research tool for studying cellular networks of protein interactions. Nucleic Acids Res 30, 303–305 (2002). 11752321 10.1093/nar/30.1.303 PMC99070

[b12] Shannon P. *et al.* Cytoscape: a software environment for integrated models of biomolecular interaction networks. Genome Res 13, 2498–2504 (2003). 14597658 10.1101/gr.1239303 PMC403769

[b13] Smoot M. E., Ono K., Ruscheinski J., Wang P. L. & Ideker T. Cytoscape 2.8: new features for data integration and network visualization. Bioinformatics 27, 431–432 (2011). 21149340 10.1093/bioinformatics/btq675 PMC3031041

[b14] Jupiter D., Chen H. & VanBuren V. STARNET 2: a web-based tool for accelerating discovery of gene regulatory networks using microarray co-expression data. BMC Bioinformatics 10, 332 (2009). 19828039 10.1186/1471-2105-10-332 PMC2765977

[b15] Jupiter D. C. & VanBuren V. A visual data mining tool that facilitates reconstruction of transcription regulatory networks. PLoS One 3, e1717 (2008). 18320038 10.1371/journal.pone.0001717 PMC2248622

[b16] Takahashi K. *et al.* Induction of pluripotent stem cells from adult human fibroblasts by defined factors. Cell 131, 861–872 (2007). 18035408 10.1016/j.cell.2007.11.019

[b17] Yu J. *et al.* Induced pluripotent stem cell lines derived from human somatic cells. Science 318, 1917–1920 (2007). 18029452 10.1126/science.1151526

[b18] Kwong L. N. *et al.* Oncogenic NRAS signaling differentially regulates survival and proliferation in melanoma. Nat Med 18, 1503–1510 (2012). 22983396 10.1038/nm.2941 PMC3777533

[b19] Wheeler D. L. *et al.* Database resources of the National Center for Biotechnology Information. Nucleic Acids Res 29, 11–16 (2001). 11125038 10.1093/nar/29.1.11 PMC29800

[b20] Arvestad L. RefSense, <https://github.com/arvestad/RefSense> (2012).

