# Supplementary material for: Managing biological complexity across orthologs with a visual knowledgebase of documented biomolecular interactions
Source: Sci Rep. 2012 Dec 20;2:1011. doi: 10.1038/srep01011 (PMC3527363; doi:10.1038/srep01011)
Supplement: Supplementary Information — Supplementary Figures [file srep01011-s1.pdf]

Managing biological complexity across orthologs with a visual knowledgebase of documented biomolecular interactions
Vincent VanBuren and Hailin Chen

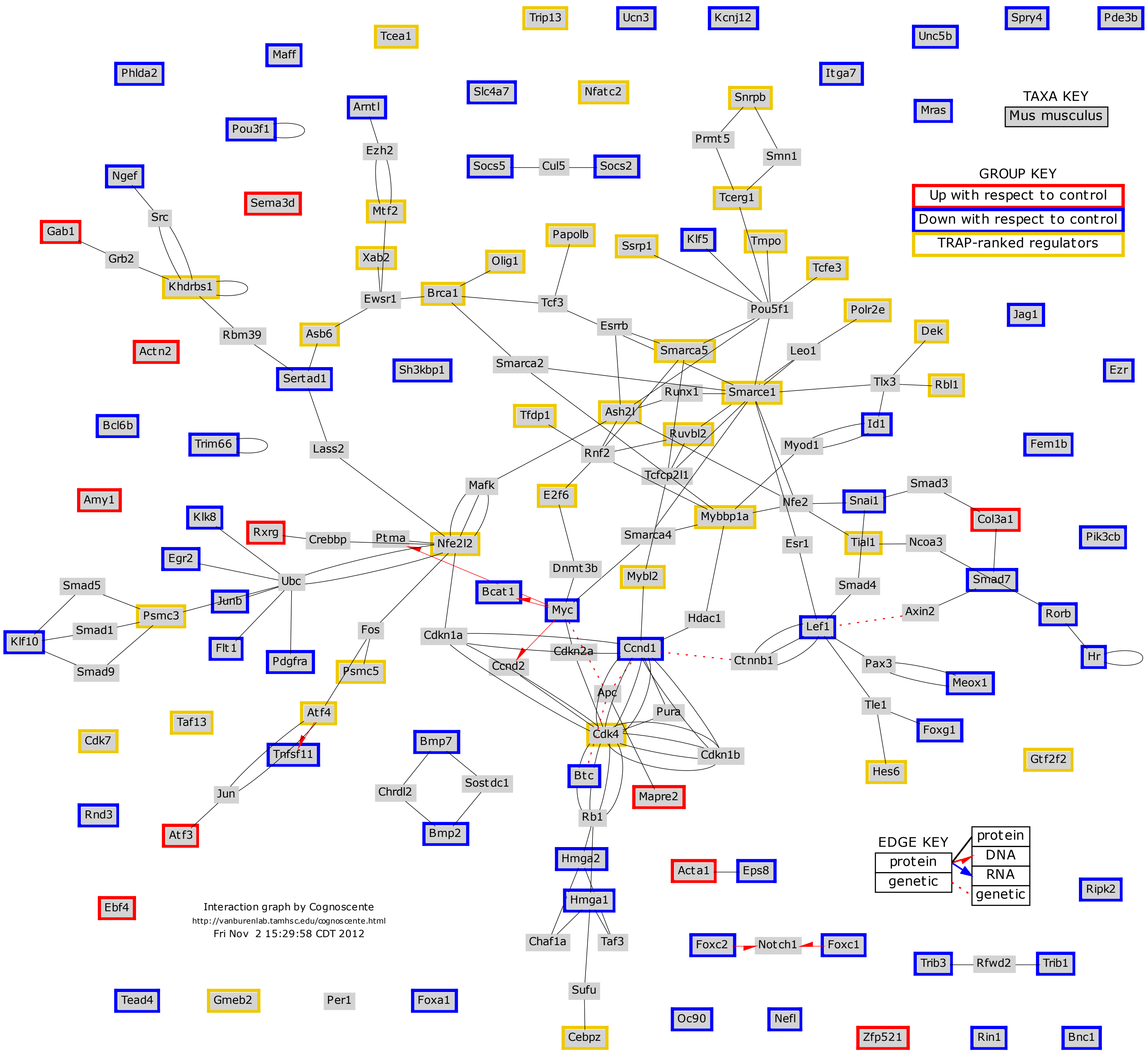

Supplementary Figure S1. Example of high-content data analysis with **Cognoscente**. Expressed genes that were up or down with respect to control, and genes identified as regulators from Kwong and colleagues' recent melanoma study[18] were queried by group in **Cognoscente**, parameterized by **Radius = 0 with intermediates**.

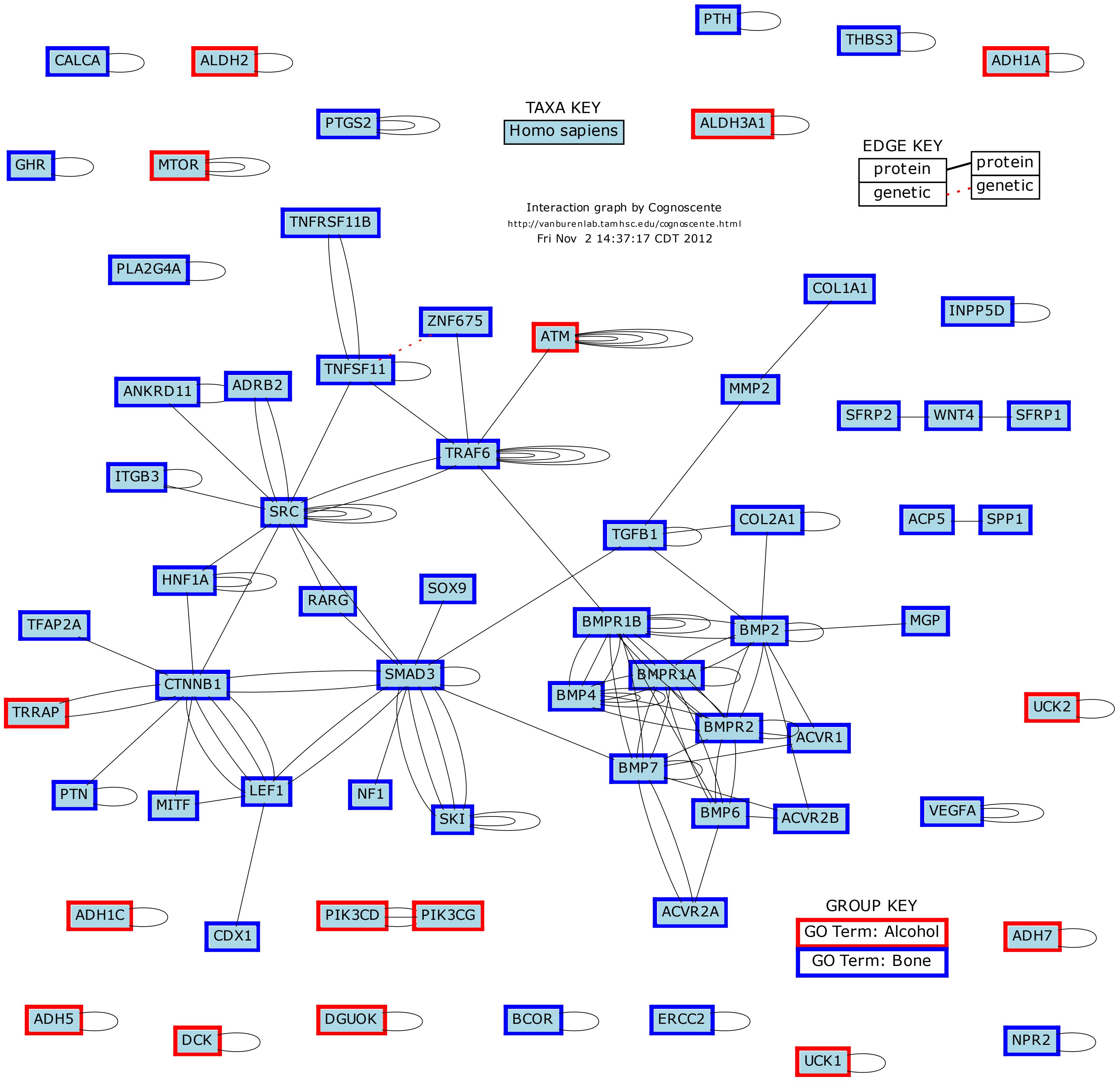

Supplementary Figure S2. Example of constructing an arbitrary interactome with **Cognoscente**. This shows the results of a query of all human genes annotated with a Gene Ontology (GO) term that contains the word “alcohol”, paired with second grouping of all human genes annotated with a GO term containing the word “bone”. This query was parameterized with **Radius = 0**.

Managing biological complexity across orthologs with a visual knowledgebase of documented biomolecular interactions

Vincent VanBuren and Hailin Chen

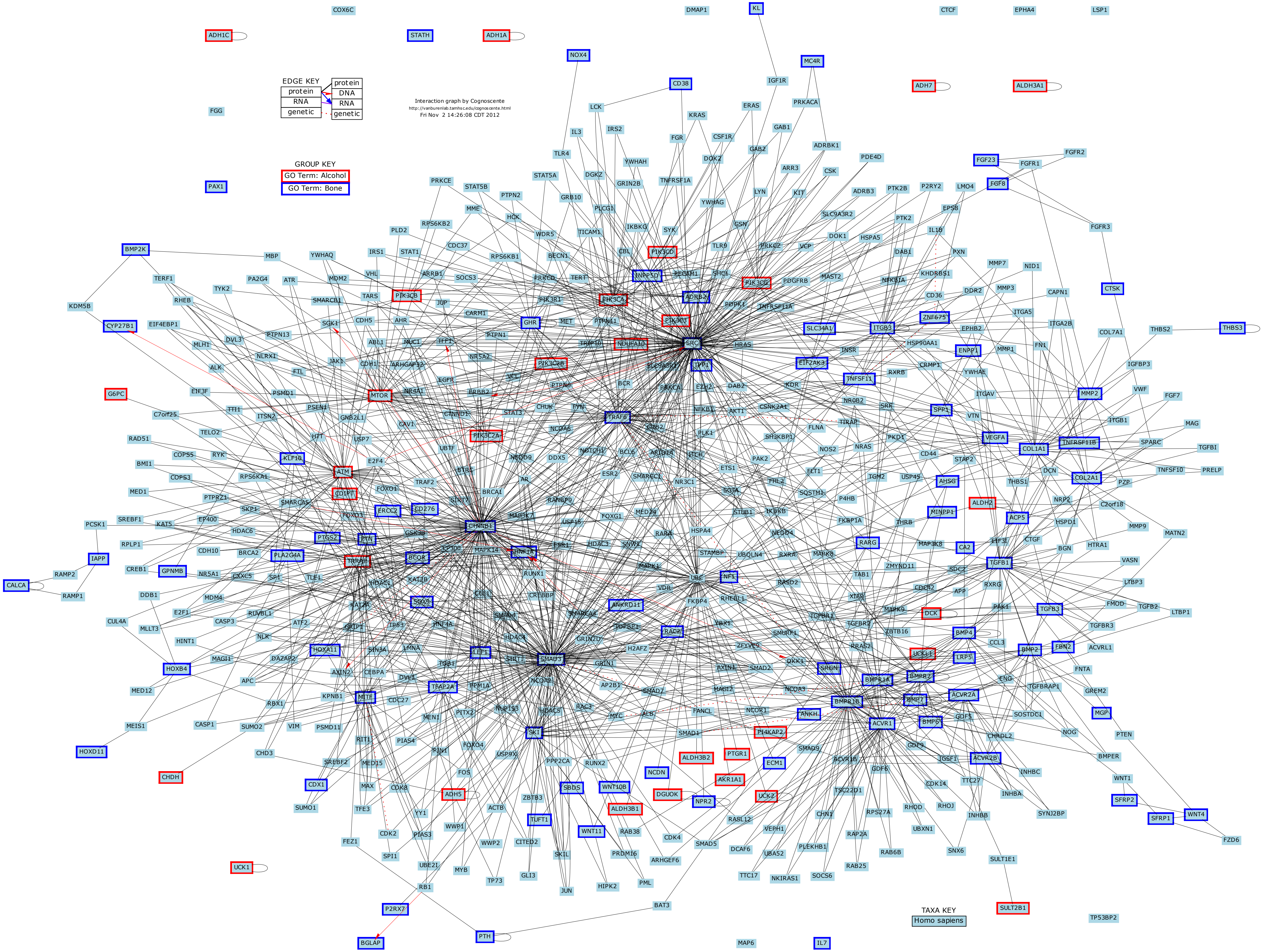

Supplementary Figure S3. Extended example of constructing an arbitrary interactome with **Cognoscente**. This graph is the result of the same query as in **Supplementary Fig. 2**, but this query was parameterized with **Radius = 0 with intermediates**.
